# The Feasibility Study of Hypofractionated Radiotherapy with Regional Hyperthermia in Soft Tissue Sarcomas

**DOI:** 10.3390/cancers13061332

**Published:** 2021-03-16

**Authors:** Mateusz Jacek Spałek, Aneta Maria Borkowska, Maria Telejko, Michał Wągrodzki, Daria Niebyłowska, Aldona Uzar, Magdalena Białobrzeska, Piotr Rutkowski

**Affiliations:** 1 Department of Soft Tissue/Bone Sarcoma and Melanoma, Maria Sklodowska-Curie National Research Institute of Oncology, 02-781 Warsaw, Poland; aneta.borkowska@pib-nio.pl (A.M.B.); piotr.rutkowski@pib-nio.pl (P.R.); 2 Department of Radiotherapy, Maria Sklodowska-Curie National Research Institute of Oncology, 02-781 Warsaw, Poland; maria.telejko@pib-nio.pl (M.T.); daria.niebylowska@pib-nio.pl (D.N.); aldona.lesniewska-mazur@pib-nio.pl (A.U.); magdalena.bialobrzeska@pib-nio.pl (M.B.); 3 Department of Pathology and Laboratory Medicine, Maria Sklodowska-Curie National Research Institute of Oncology, 02-781 Warsaw, Poland; michal.wagrodzki@pathologist.cc

**Keywords:** sarcoma, radiotherapy, neoadjuvant therapy, hypofractionated radiotherapy, thermotherapy, hyperthermia

## Abstract

**Simple Summary:**

The recommended management of marginally resectable or unresectable soft tissue sarcomas is an attempt of neoadjuvant therapy. The use of neoadjuvant chemotherapy is limited in low-grade tumors, sarcomas with chemoresistant pathology or in unfit patients. There is a growing evidence on hypofractionated radiotherapy in soft tissue sarcomas, but its efficacy may be limited by radioresistance that is frequently associated with chemoresistance. Regional hyperthermia is a potent and minimally invasive radiosensitizer. We aimed to investigate the feasibility of moderately hypofractionated radiotherapy combined with regional hyperthermia in aforementioned clinical situations. Our findings indicate that proposed combination is feasible while maintaining good short-term local efficacy and tolerance. It could serve as a basis for further studies on radiotherapy with hyperthermia in soft tissue sarcomas.

**Abstract:**

Introduction: Management of marginally resectable or unresectable soft tissue sarcomas (STS) in patients who are not candidates for neoadjuvant chemotherapy due to chemoresistant pathology or contraindications remains a challenge. Therefore, in these indications, we aimed to investigate a feasibility of 10x 3.25 Gy radiotherapy combined with regional hyperthermia (HT) that could be followed by surgery or 4x 4 Gy radiotherapy with HT. Materials and methods: We recruited patients with locally advanced marginally resectable or unresectable STS who (1) presented chemoresistant STS subtype, or (2) progressed after neoadjuvant chemotherapy, or (3) were unfit for chemotherapy. The primary endpoint was the feasibility of the proposed regimen. Results: Thirty patients were enrolled. All patients received the first part of the treatment, namely radiotherapy with HT. Among them, 14 received the second part of radiotherapy with HT whereas 13 patients underwent surgery. Three patients did not complete the treatment protocol. The feasibility criteria were fulfilled in 90% of patients. Two patients developed distant metastases. One patient died due to distant progression. One patient developed rapid local recurrence after surgery. Conclusions: Hypofractionated radiotherapy with HT is a feasible treatment for marginally resectable or unresectable STS in patients who are not candidates for chemotherapy. Results of this clinical trial support the further validation of RT and HT combinations in STS.

## 1. Introduction

The recommended management of marginally resectable or unresectable soft tissue sarcomas (STS) is an attempt of neoadjuvant therapy, namely radiotherapy (RT) and chemotherapy [1,2,3]. Neoadjuvant chemotherapy was a preferred approach in the vast majority of European sarcoma tertiary centers as per the expert survey performed by the European Organization for Research and Treatment of Cancer [4]. However, the most effective but toxic chemotherapy regimens used in STS may be not suitable for elderly patients or those with significant comorbidities [5]. Moreover, low-grade STS and selected histopathological STS subtypes are considered chemoresistant [6,7,8,9,10,11,12]. Another approach could be the use of high-dose definitive RT. In a large cohort analysis on RT for unresectable STS, total dose and tumor size influenced on local control, disease-free survival and overall survival [13]. This study identified a threshold dose of 63 Gy. However, delivery of such doses to large volume with extensive margins may lead to increase of RT-related toxicity as per results of the aforementioned analysis.

The solution may be the introduction of hypofractionated regimens. Alpha/beta ratio of STS, especially low-grade, is presumably low or very low [14,15]. Therefore, a higher dose per fraction may enable better tumor control with lower total equivalent dose in 2-Gy fractions (EQD2) and the similar toxicity profile [16]. This hypothesis was validated in several early-phase clinical trials with hypofractionation for STS [17,18,19,20,21,22,23,24]. Unfortunately, chemoresistance is frequently associated with radioresistance; thus, hypofractionated RT alone may not provide satisfactory local control [25,26]. The efficacy of RT could be additionally increased by using various methods that may overcome radioresistance. Hyperthermia (HT) is known as a potent radiosensitizer that enhance the cell-killing effect of RT and chemotherapy [27,28]. Focused heat directly damages tumor cells that are more heat-sensitive than surrounding tissues. It also indirectly intensifies RT damage by increasing oxygenation and inducing apoptosis, instability of the cell membrane, and dysregulation of proteins, including DNA repair enzymes [29]. Despite supporting evidence from preclinical studies, the addition of HT to standard neoadjuvant therapy was rarely a matter of prospective clinical trials [30,31,32]. The only randomized phase 3 clinical trial showed increased survival, as well as local progression-free survival among patients with locally advanced STS who received regional HT additionally to neoadjuvant chemotherapy [33,34]. Such an evidence for RT with HT does not exist. However, this combination provided encouraging results in management of melanomas that are also considered as chemo- and radioresistant tumors [35].

Therefore, we hypothesized that hypofractionated RT combined with regional HT is a feasible method of treatment of patients with STS who are not candidates for chemotherapy. We aimed to design feasible and flexible regimen that provides benefits from both hypofractionation and HT, as well as it can be applied for marginally resectable and unresectable tumors. Thus, we proposed two-week regimen of 32.5 Gy in 10 fractions with four HT sessions that could be followed by surgery or the second part of RT with HT.

Hence, we report the results of the clinical trial hypofractionated RT with regional HT for STS.

## 2. Materials and Methods

We conducted a prospective proof-of-concept phase II, open-label, single-arm clinical trial (NCT03989596). Ethical approval for this study was obtained from the Institutional Ethics committee of Maria Sklodowska-Curie National Research Institute of Oncology in June 2018, approval number 35/2018.

### 2.1. Inclusion Criteria

We recruited adult patients with locally advanced marginally resectable or unresectable STS localized to the extremities, trunk wall and pelvis who were not candidates for neoadjuvant chemotherapy. Resectability, chemoresistance, and/or non-eligibility for chemotherapy (for example, due to patient’s comorbidities) were discussed individually in each case during the sarcoma multidisciplinary tumor board (MTB).

The major institutional criteria of marginal resectability and unresectability included extracompartmental extension of the tumor, involvement of the bone or major vessels that may require vascular reconstruction, an extension of the tumor through natural foramina, or technical difficulties with resectability due to the tumor volume or its anatomical localization.

Patients who were not appropriate candidates for chemotherapy included those with chemoresistant STS or unfit to tolerate such a treatment as per MTB decision. Chemoresistance was defined as clinical or radiological local progression of primary tumor on neoadjuvant chemotherapy or the diagnosis of potentially chemoresistant STS (low-grade STS, epithelioid sarcoma, clear cell sarcoma, alveolar soft part tissue sarcoma, solitary fibrous tumor).

All patients were 18 or older and had Eastern Cooperative Oncology Group (ECOG) performance status 0 to 2. All patients provided written informed consent as approved by the Institutional Ethics Committee.

### 2.2. Exclusion Criteria

Patients with distant metastases, lymph node involvement or contraindications to RT or HT were excluded. Excluded pathological diagnoses were Ewing sarcoma, osteogenic sarcoma, embryonal, or alveolar rhabdomyosarcoma, and aggressive fibromatosis. Neither prior RT within or close to the currently planned target volume (PTV) nor second active malignancy were permitted.

### 2.3. Treatment Schedule

After a screening, which consists of local assessment of primary tumor in magnetic resonance imaging (MRI) or computed tomography (CT), biopsy or central pathological assessment of previously taken tumor sample, physical examination, exclusion of distant metastases in staging imaging, and case analysis at the MTB meeting, a patient received 32.5 Gy in ten fractions with regional HT twice a week within two weeks. The response analysis in CT or MRI and toxicity assessment were performed after at least six weeks.

**Figure 1:**
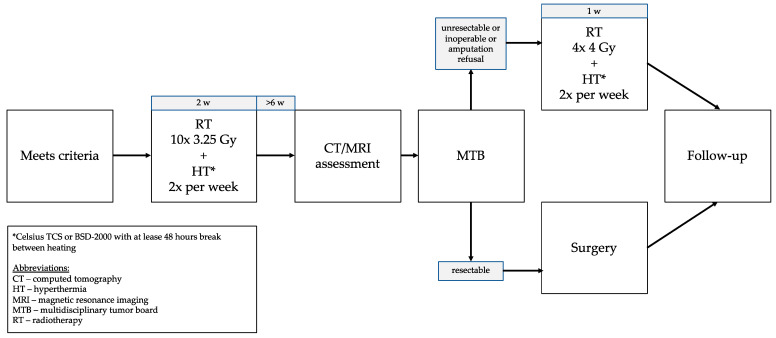
Treatment schedule.

During the second MDT meeting, final decisions about resectability and operability were made. In the case of resectability, operability, or consent for amputation if required, a patient was referred to surgery. Otherwise, the patient received a second part of local treatment which consisted of 16 Gy in four fractions with regional HT twice a week within one week. The treatment schedule was presented in Figure 1.

### 2.4. Radiotherapy

Previous clinical trials with moderate hypofractionation combined with chemotherapy showed good local efficacy and favorable toxicity profile of such treatment. The most common fractionation regimens comprised of 28–35 Gy in eight to ten fractions combined with anthracycline-based chemotherapy. We used the alpha/beta ratio for STS of 4 Gy, in alignment with other studies. Then, we decided to use 32.5 Gy in ten fractions. That translated into equivalent dose in 2-Gy fractions (EQD2) of 39.3 Gy. The addition of four HT sessions should compensate the lack of chemotherapy and lower EQD2 than in conventionally fractionated regimens; thus, we expected high local control. Moreover, there is theoretical dependence between higher total dose and a risk of RT-related toxicity, mostly wound complications. Thus, investigated regimen with lower EQD2 should provide a substantial benefit for the patients with locally advanced STS who are not candidates for chemotherapy.

In the case of unresectability, inoperability or refusal to amputation if limb-sparing surgery was not possible, the patient received the second part of RT with HT. It consisted of 16 Gy in four fractions combined with two HT sessions. That translated into higher dose intensity and EQD2 of 21.3 Gy. Then, the total EQD2 from the whole RT with HT was 60.6 Gy.

Delineation and treatment planning was performed by a team experienced in STS. The immobilization and the application of bolus was selected on a case-by-case basis. The gross tumor volume (GTV) was contoured on planning CT fused with contrast-enhanced MRI or diagnostic contrast-enhanced CT. The general rule was to create clinical target volume (CTV) by expanding GTV at least 2 cm in each direction, and, in extremity STS, at least 4 cm longitudinally. Nevertheless, due to the variety of clinical situations, the final choice of CTV was based on the benefit-risk assessment and opinion of at least two other radiation oncologists. In the case of second part RT with HT, it was allowed to reduce CTV to 1.5 cm in each direction. Then, CTV was adapted to anatomical borders of tumor spread (i.e., bones, fascias, or vital organs). All delineated target volumes and organs at risk were reviewed by another radiation oncologist. Planning target volume (PTV) was created by expanding CTV, adding safety margins (0.5–1.0 cm).

We used three-dimensional RT techniques with daily image guidance (cone beam computed tomography or planar kilovoltage) to deliver the prescribed dose. Techniques with dose intensity modulation were preferred over static RT. The dose was prescribed on mean PTV.

### 2.5. Hyperthermia

We used two regional HT systems, Celsius TCS and BSD-2000. The choice of the equipment for heat delivery in each case was based on tumor and patient-related factors, such as tumor localization or weight and height. BSD-2000 was preferred in the case of deeply seated abdominal and pelvic tumors or tumors localized to lower limbs; however, due to the properties of the Sigma-Eye applicator, it was not possible to use it in the majority of obese and overweight patients. Celsius TCS system was used in the other situations. The final decision was made by a radiation oncologist after discussion with radiation therapists specialized in HT.

Celsius TCS system uses the changeable two-electrode and water bolus system that allow homogeneous temperature development within the heated volume. The heat energy is generated by electromagnetic waves of 13.56 MHz to transfer energy based on the principle of capacitive coupling. More detailed data are provided in the manufacturer’s site [36]. The disadvantage of the Celsius TCS system include lack of tumor temperature control; however, we used pre-defined treatment protocols tailored to anatomical site and patient’s parameters to ensure proper delivery of necessary heat energy. The choice of treatment protocol was discussed within the HT team, taking into account tumor site, volume, and comorbidities.

BSD-2000 3D system uses 24 antennas surrounding patient’s body that generate electromagnetic waves at the frequency range of 75 to 140 MHz to deliver heat energy. The energy is focused on heated volume using dedicated software. More detailed data are provided in the manufacturer’s site [37]. We aimed to reach temperature within the treated volume between 39 and 42 Celsius degree. The indirect temperature control was performed using intraluminal or skin sensors.

Institutional contraindications to HT included implanted medical devices or objects (for example pacemakers, stabilizers, prostheses), significant tumor-related pain that cannot be controlled with medications, severe cardiac or pulmonary diseases, uncontrolled hypertension, myocardial infarction or cerebrovascular incident <6 months ago, pregnancy or breastfeeding.

HT was performed by a dedicated team of radiation therapists. Thermotolerance and all protocols deviations were strictly monitored and reported. The treatment was applied twice a week, with a minimum 48-h gap between sessions. The patient received RT fraction within one hour after each HT session.

### 2.6. Assessment of Response and Toxicity

Radiological response was assessed according to the Response Evaluation Criteria in Solid Tumors (RECIST), version 1.1. RT and HT toxicities were assessed according to the Common Terminology Criteria for Adverse Events v 5.0 (CTCAE). Acute toxicity was defined as from start of the RT with HT to 90 days after treatment completion. Late toxicity was defined as at any time after 90 days.

### 2.7. Follow-Up

The patients are followed-up according to the national and international guidelines. The minimum is a first visit 30 days after treatment completion to assess RT toxicity or wound healing and then every three months in the first two years, then twice a year up to the fifth year, and once a year thereafter. All patients are followed for evidence of local recurrence or distant metastases using CT or X-ray of the chest, and MRI or CT of the treated site.

### 2.8. Primary Endpoint

In this proof-of-concept study, the primary endpoint was the protocol feasibility that comprised treatment tolerance and compliance. The study would be terminated earlier in the case of unacceptable toxicity of treatment defined as the frequency of occurrence of grade 3 or higher acute toxicity according to CTCAE in over 40% of the treated patients.

The intervention would be deemed feasible if it meets safety rule and at least 80% of participants fulfil all the criteria:

- Able to finish all planned treatment according to the protocol (intention-to-treat principle); a permanent treatment termination regardless of the reason, including consent withdrawal, lost to follow-up or disease progression, was treated as the protocol failure.
- Able to tolerate HT; reduction of delivered heat energy or temporary breaks were allowed, but permanent discontinuation was treated as the protocol failure.
- Able to tolerate hypofractionated RT without unplanned breaks.

Using the Wilson method for calculating confidence intervals for proportions, the exact 95% confidence interval for an estimated feasibility proportion of 80% (23 of 30 patients) does not include (60–80%) a value of 50%. Thus, for a sample size of 30 patients, a feasibility of 80% is above chance level performance (50%).

### 2.9. Secondary Endpoints

The secondary endpoints were one-year local control, one-year sarcoma-specific survival (SSS), one-year progression-free survival (PFS) and rate of late toxicities. Local control was assessed by calculating time to local progression (TTLP) and local progression-free survival (LPFS).

### 2.10. Statistical Considerations

Statistical analyses were performed using R version 3.6.3 software (R Foundation for Statistical Computing, Vienna, Austria) and jamovi version 1.2 software (The jamovi project, Sydney, Australia).

The one-sample proportions test and the one-sided alternative were used to assess whether the feasibility proportion is larger than 50%. Median follow-up was estimated by Kaplan–Meier analysis with the reversed meaning of the status indicator. TTLP was calculated from the day of surgery or start of the last part of RT with HT to the last follow-up (censored), death without local progression (censored) or confirmed local progression. LPFS was calculated from the day of surgery or start of the last part of RT with HT to the last follow-up (censored), confirmed local progression, or death. SSS was calculated from the enrollment to the last follow-up (censored), death from STS, or death from other reasons (censored). PFS was calculated from the enrollment to the last follow-up (censored), disease progression or death. The Kaplan–Meier method was used to estimate survival. All *p*-values < 0.05 were considered significant.

## 3. Results

### 3.1. Patient and Tumor Data

Between June 2018 and September 2020, 30 patients were enrolled. The most frequent pathological diagnosis was solitary fibrous tumor (23%). The vast majority of STS were low grade tumors (73%). Over one third of the treated tumors developed in the pelvic area (37%). The median largest tumor dimension was 9.8 cm. At the MTB, 17 tumors were classified as marginally resectable, eight as locally unresectable but with possible amputation of the extremity and five as unresectable regardless the extent of surgery. The patient and tumor characteristics are presented in Table 1. Detailed patient, tumor and treatment data are available in Table S1.

### 3.2. Feasibility and Applied Treatment

Using intention-to-treat principle, all patients received the first part of RT with HT (BSD-2000, *n* = 7; Celsius-TCS, *n* = 23), whereas all but three received the second part of treatment, namely surgery or the second part of RT with HT (BSD-2000, *n* = 3; Celsius-TCS, *n* = 11). One patient received three out of four planned HT sessions due to HT equipment breakdown; however, this event was not considered as treatment failure. Thus, the feasibility proportion was 90% (27/30), with a 95% confidence interval (CI) equal to 76–100%. The feasibility proportion was greater than 50% with a *p*-value < 0.001.

The first patient who did not complete the protocol refused further cancer treatment due to the deterioration of performance status caused by comorbidities. The second one developed multiple lung metastases after RT with HT and was referred to palliative treatment. The third patient was lost to follow-up after the first RT with HT and did not answer phone calls.

**Figure 2:**
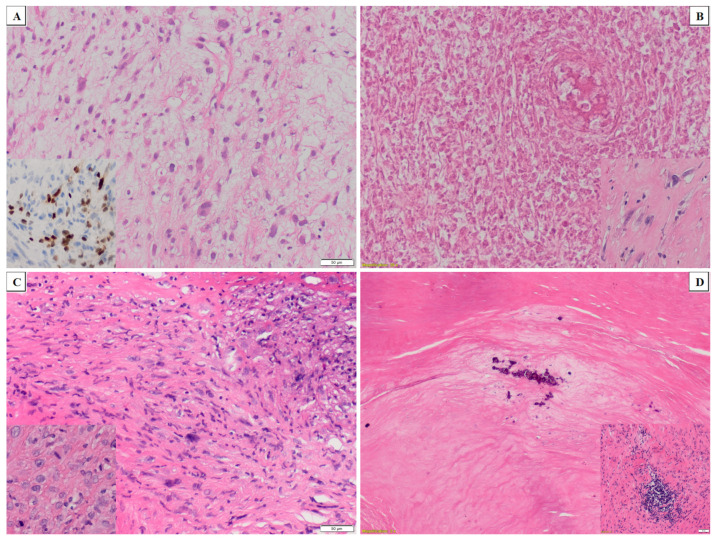
Histological response to the treatment: (**A**) A pleomorphic rhabdomyosarcoma before treatment confirmed by the myogenin expression (clone F5D, Daco, inlet); (**B**) diffuse necrotic changes in the pleomorphic rhabdomyosarcoma after treatment with only single, degenerated neoplastic cells present (less than 1% of tumoral tissue, inlet); (**C**) biopsy of a high-grade, undifferentiated spindle cell and focally pleomorphic sarcoma with brisk mitotic activity (inlet); (**D**) diffuse sclerosis with focal calcifications and scattered lymphocytic infiltrates in more fibrotic areas (inlet) in the undifferentiated sarcoma after treatment.

Among patients who completed the whole protocol, 13 underwent surgery while 14 were referred to the second part of RT with HT. The median GTV, CTV, and PTV during the first part of RT with HT were 299 cm^3^, 1586 cm^3^, and 2153 cm^3^, respectively. The corresponding median values of target volumes in the second part of the local treatment were 228 cm^3^, 638 cm^3^, and 1041 cm^3,^ respectively. Among patients who underwent surgery, microscopically negative margins were achieved in 13/15 patients (87%). The summary is presented in Table 2. Interestingly, complete or almost complete pathological responses according to the European Organization for Research and Treatment of Cancer-Soft Tissue and Bone Sarcoma Group recommendations were found in three and one postoperative specimens, respectively (Figure 2).

RT was well-tolerated. The analysis of RT-related toxicities is presented in Table 3. The full analysis of late toxicities will be possible after longer follow-up.

The tolerance of HT was acceptable. The most common adverse event during heating was an unpleasant sensation of high heat (43.3% of patients). HT-related adverse events were presented in Table 4. We did not observe any HT-related toxicity after heating. Full data regarding used electrodes, applicators sensors and protocols, temperature range, delivered energies, heating times, detailed description of adverse events and thermotolerance are available in Table S2.

### 3.3. Local Control and Survival

Median follow-up time was 13 months (interquartile range: 10–17 months). At the moment of analysis, 29 patients were alive (97%). Rapid local recurrence treated with extremity amputation was observed in one patient who underwent RT with HT followed by R1 surgery. Distant metastases were diagnosed in two patients, being the cause of death in one of them. One-year TTLP and LPFS were 97% and 93%, respectively. One-year SSS and PFS were 97% and 88%, respectively. Survival curves are available in Figure S1.

## 4. Discussion

Our study prospectively examined the feasibility of moderately hypofractionated RT combined with regional HT that was performed using two HT-dedicated devices. Despite large volumes of treated tumors (median 9.8 cm) and anatomically challenging localizations, such as pelvis (37%), the feasibility criteria were fulfilled by 90% of enrolled patients. There is lack of other clinical trials that investigate the combination of RT with HT in STS, thus, the comparison of results is not possible.

Preliminary results for local efficacy, occurrence of acute toxicity and ratio of wound complications are similar to those achieved in other series with hypofractionated RT [17,20]. Despite relatively low EQD2 of the proposed regimen, we observed four very good pathological responses among patients who underwent surgery (31%) and partial radiological responses among patients who received the second part of RT with HT (57%). It indicates vast radiosensitizing effect of HT. Any grade HT-related adverse events occurred in 19/30 patients (63%), leading to decrease of HT intensity (power reduction or shortened heating time) in 11 cases (Table S2). In turn, we did not observe any serious adverse event after heating or toxicity that could be detected in physical examination, such as blisters or burns. Mild and moderate HT-related adverse events were also reported in the largest study on neoadjuvant chemotherapy with HT for STS that was published by Issels et al. [34]. Then we can assume that HT is well-tolerated treatment in comparison to other modalities used in STS management.

Until the moment of the analysis, we observed few late toxicities (Table S1). All but one of the late toxicities affected patients who underwent the second part of RT with HT. Only one patient who underwent RT for pelvic STS experience serious late toxicity, namely grade 3 colonic bleeding due to massive telangiectasia that required blood transfusion and laser treatment. Nevertheless, the full impact of investigated regimen on long-term local efficacy and occurrence of late toxicities will be assessed after longer follow-up.

The study has several limitations. The first weakness is the lack of clear definitions of marginal resectability and unresectability in STS. In our study, resectability was assessed by surgical oncologists experienced in STS who used institutional and international criteria during MTB meeting [38].

Second, one may ask whether the definitive RT is appropriate method for patients with low-grade STS localized to extremities that can be removed with amputation. However, after amputation refusal, RT seems to be a better treatment option than chemotherapy or observation only. The risk of distant metastases in this group remains low [39,40]. In the case of local progression after RT, amputation still remains an option. Importantly, in our group none of the patients locally progressed after two parts of RT with HT in short-term follow-up.

Third, our study group is heterogenous. The patients were diagnosed with numerous STS subtypes and presented different clinical situations. Nevertheless, we aimed to design a flexible regimen that covers challenges related to STS treatment and could be used in various indications.

Finally, data regarding proper application of HT in STS are greatly limited. The choice of HT equipment, protocols, energies, and other aspects of heating was based upon HT team decision rather than on objective criteria. However, such recommendations do not exist. Moreover, we were only able to estimate the real intratumoral temperature during HT. To maintain feasibility and tolerability we did not use risky invasive intratumoral temperature monitoring. We used pre-defined protocols for Celsius TCS without temperature control due to unavailability of dedicated temperature sensors. In the case of BSD-200, skin and intraluminal sensors enable only indirect measurements. The solution could be the introduction of magnetic resonance thermometry or other methods of temperature measurement in further investigations [41].

The study does not provide a new standard of treatment, neither confirms long-term efficacy of the proposed regimen. This trial rather suggests that moderately hypofractionated RT could be combined with regional HT while maintaining treatment compliance, short-term local efficacy and favorable toxicity profile in challenging clinical situations with locally advanced STS. The results could serve as the basis for the development of new studies on RT with HT in STS.

## 5. Conclusions

Moderately hypofractionated RT with regional HT seems to be feasible method of neoadjuvant or definitive treatment for marginally resectable or unresectable locally advanced STS in patients who are not candidates for chemotherapy due to chemoresistance or contraindications. Preliminary observations suggest good tolerance and no decrease in local efficacy of such treatment; however, the optimal application of HT remains a challenge. Results of this clinical trial support the further validation of RT and HT combinations in STS.

## Figures and Tables

**Table 1:** Patient and tumor characteristics.

Characteristic		Value
Age at the enrollment	Median	69.5
	Interquartile range	52–74.8
Largest tumor dimension	Median	9.8 cm
	Interquartile range	7.7–13.5 cm
		**Number of patients (%)**
Sex	Female	17 (56.7)
	Male	13 (43.3)
Tumor grade	1	22 (73.3)
	2	3 (10)
	3	4 (13.3)
	Not assessed *	1 (3.3)
Pathological diagnosis (biopsy)	Solitary fibrous tumor	7 (23.3)
	Leiomyosarcoma	5 (16.7)
	Sarcoma not otherwise specified	4 (13.3)
	Undifferentiated pleomorphic sarcoma	3 (10)
	Myxoid liposarcoma	2 (6.7)
	Myogenic sarcoma	1 (3.3)
	Pleomorphic rhabdomyosarcoma	1 (3.3)
	Sclerosing epithelioid fibrosarcoma	1 (3.3)
	Alveolar soft part tissue sarcoma	1 (3.3)
	Myxoinflammatory fibroblastic sarcoma	1 (3.3)
	Low grade fibromyxoid sarcoma	1 (3.3)
	Myxofibrosarcoma	1 (3.3)
	Well-differentiated liposarcoma	1 (3.3)
	Dedifferentiated liposarcoma	1 (3.3)
Primary tumor site	Pelvis	11 (36.7)
	Thigh	9 (30)
	Calf	4 (13.3)
	Forearm	2 (6.7)
	Arm	1 (3.3)
	Thorax	1 (3.3)
	Lumbar area	1 (3.3)
	Foot	1 (3.3)

* Alveolar soft part tissue sarcoma.

**Table 2:** Outcomes of treatment among patients who completed the protocol.

Parameter		Second Part of Radiotherapy with Hyperthermia: Best Local Response		Surgery: Surgical Margins	
		Stable Disease	Partial Response	R0	R1
**Grade**	1	5	6	8	1
	2	1	0	1	0
	3	0	1	2	1
	NA	0	1	0	0
Reason for inclusion	Chemoresistant subtype	5	6	9	1
	Progression after neoadjuvant CHT	0	1	1	0
	Unfit for CHT	1	1	1	1
Resectability	Marginally resectable	1	1	11	2
	Amputation only	4	4	0	0
	Unresectable	1	3	0	0

CHT—chemotherapy; R0—microscopically negative surgical margin; R1—microscopically positive surgical margin.

**Table 3:** Toxicity of radiotherapy.

Toxicity	Grade		
Early		Part of treatment	
		I	II
Radiation dermatitis	1	7	3
	2	4	
Edema	1	3	
Diarrhea	2	3	
Pain	1	1	
Hematuria	1	1	
Wound complications	1	1	NA
	2	2	NA
	3	2	NA
Late			
Recurrent hematuria	1	1	
Superficial soft tissue fibrosis	1	4	
Deep connective tissue fibrosis	2	1	
Lymphedema	1	1	
	2	2	
Colonic bleeding	3	1	

NA—not applicable.

**Table 4:** The summary of hyperthermia-related adverse events.

Adverse Events	Equipment	Grade *	Number of Patients (%)	Number of Hyperthermia Sessions (%)
			*n* = 30	*n* = 148
Sensation of high heat	Celsius TCS	1	5 (16.7)	10 (6.8)
		2	8 (26.7)	10 (6.8)
Pain	Celsius TCS	2	2 (6.7)	7 (4.7)
	BSD-2000	1	1 (3.3)	2 (1.4)
Inability to keep position	Celsius TCS	not applicable	4 (13.3)	4 (2.7)
Frequent breaks	Celsius TCS	not applicable	2 (6.7)	2 (1.4)
Electrode translocation	Celsius TCS	not applicable	2 (6.7)	2 (1.4)
Power reduction	Celsius TCS	not applicable	3 (10)	7 (4.7)
Heating time reduction	Celsius TCS	not applicable	9 (30)	15 (10.1)
	BSD-2000	not applicable	1 (3.3)	1 (0.7)
Temporary electrode breakdown	Celsius TCS	not applicable	1 (3.3)	1 (0.7)
Device breakdown	Celsius TCS	not applicable	1 (3.3)	1 (0.7)

* grade 1—sensation only, grade 2—required intervention, grade 3—hospitalization or medically significant, grade 4—life-threatening or urgent intervention indicated (prepared on the basis of the Common Terminology Criteria for Adverse Events).

## Data Availability

All data generated and analyzed during this study are included in this published article (and its Supplementary Information Files).

## Supplementary Materials

The following are available online at https://www.mdpi.com/2072-6694/13/6/1332/s1, Table S1: Patient Data, Table S2: Hyperthermia Data, Figure S1: Survival Curves.
