# Supplementary material for: The Feasibility Study of Hypofractionated Radiotherapy with Regional Hyperthermia in Soft Tissue Sarcomas
Source: Cancers (Basel). 2021 Mar 16;13(6):1332. doi: 10.3390/cancers13061332 (PMC8000962; doi:10.3390/cancers13061332)
Supplement: Supplementary file 1 [file cancers-13-01332-s001.zip › Figure S1.pptx]

## Slide 1
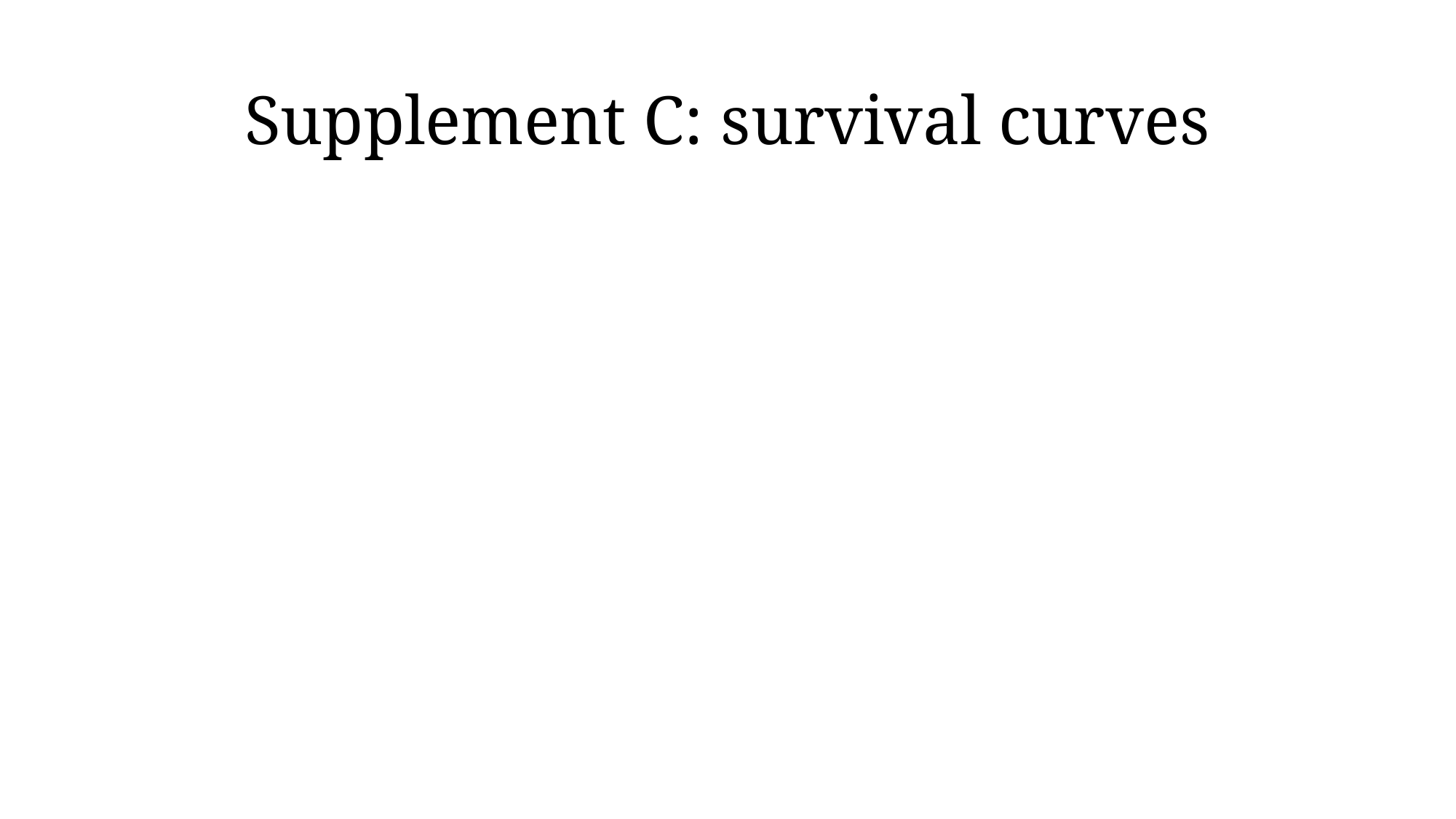

# Supplement C: survival curves

## Slide 2
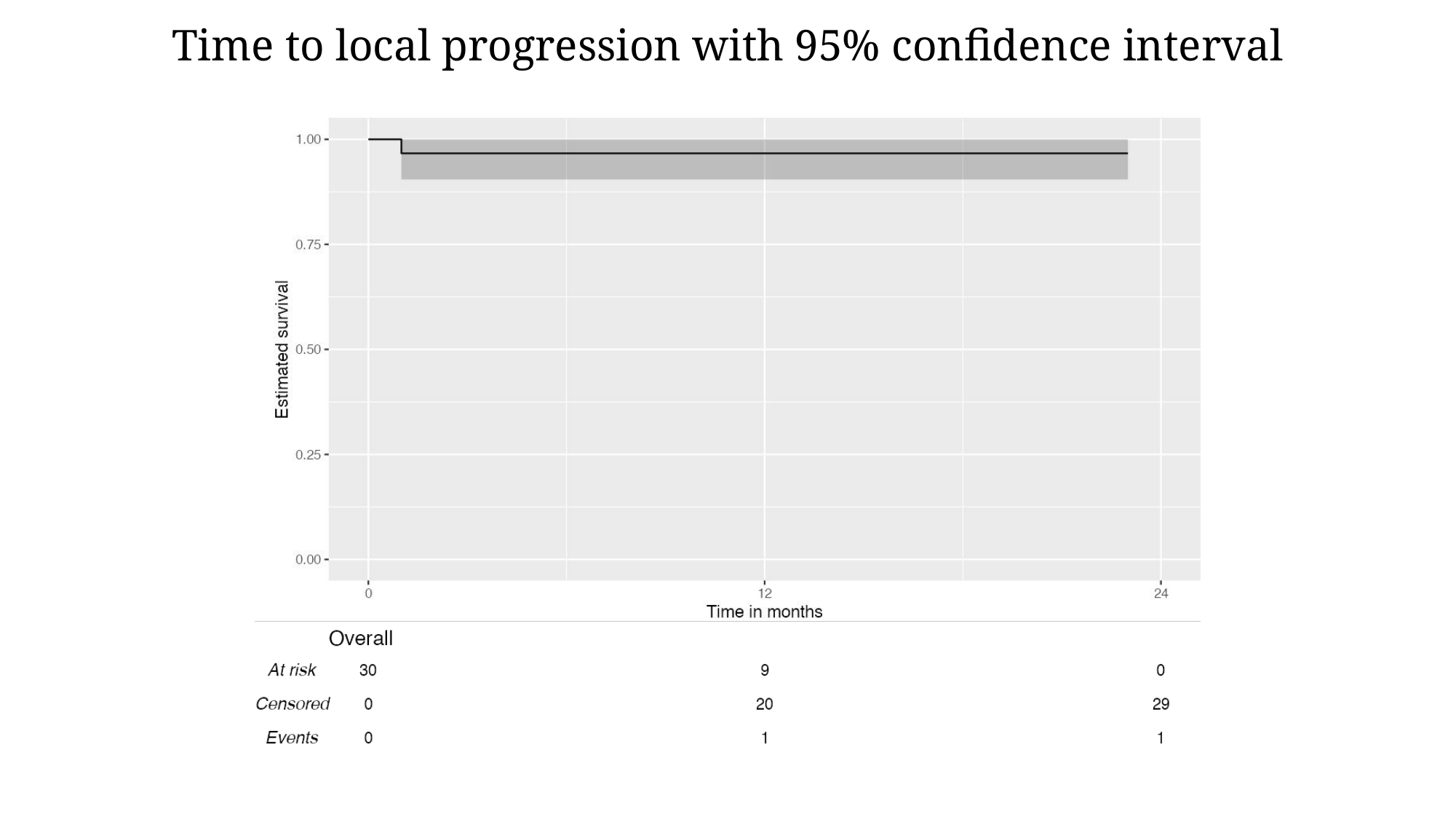

# Time to local progression with 95% confidence interval

## Slide 3
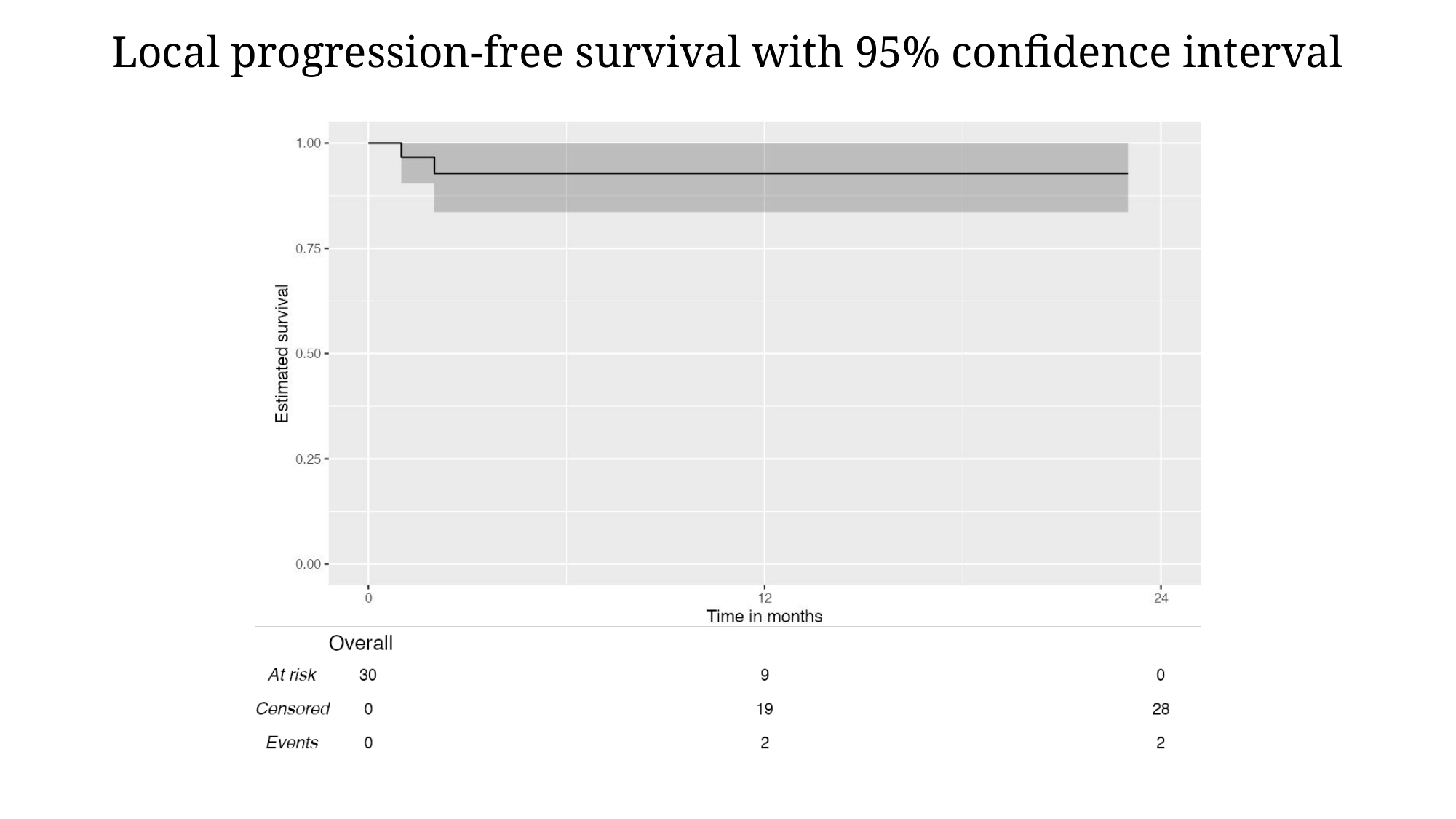

# Local progression-free survival with 95% confidence interval

## Slide 4
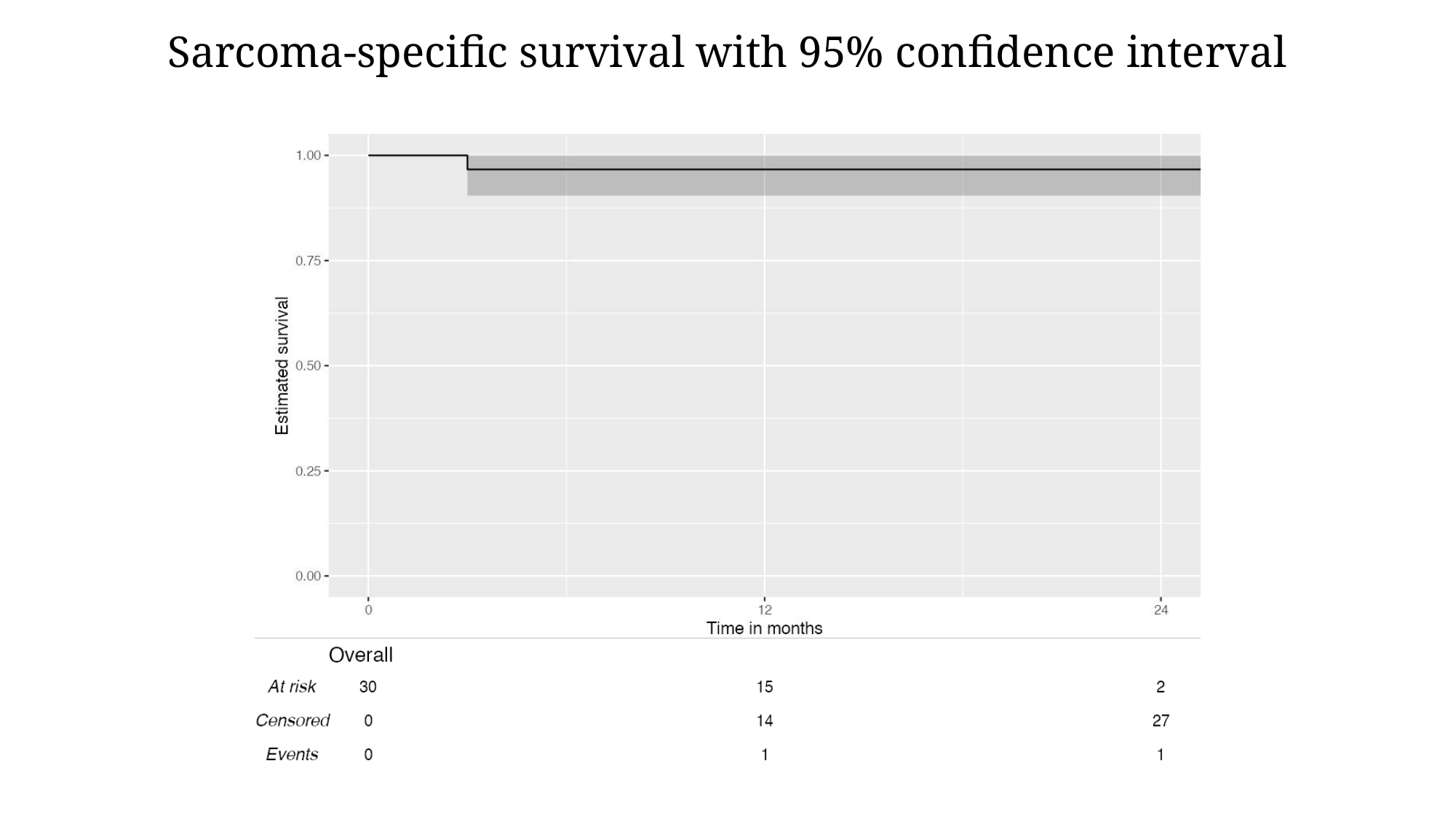

Sarcoma-specific survival with 95% confidence interval

## Slide 5
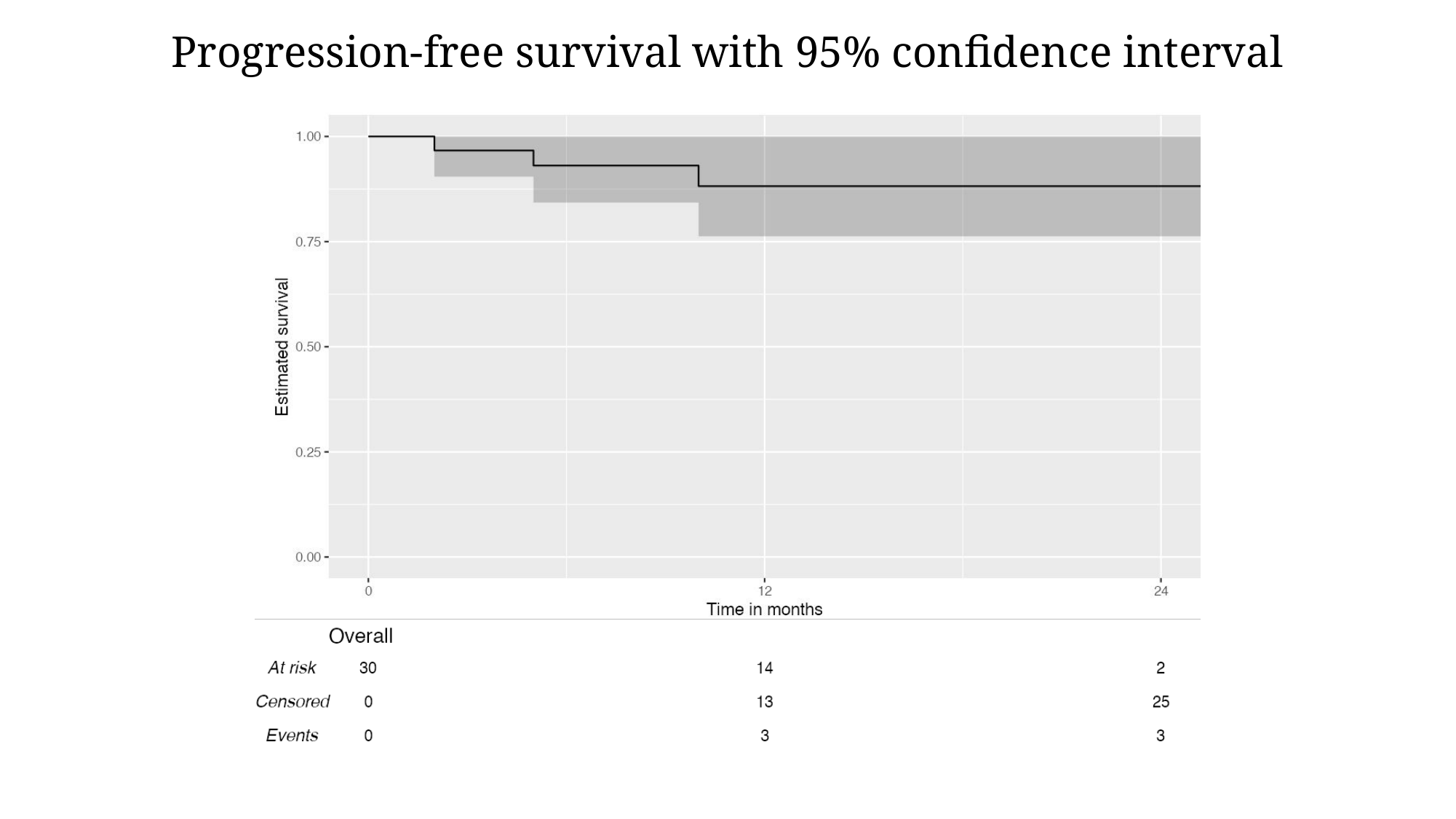

Progression-free survival with 95% confidence interval
